# Tumors with unmethylated *MLH1* and the CpG island methylator phenotype are associated with a poor prognosis in stage II colorectal cancer patients

**DOI:** 10.18632/oncotarget.13441

**Published:** 2016-11-12

**Authors:** Tao Fu, Yanliang Liu, Kai Li, Weiwei Wan, Emmanouil P. Pappou, Christine A. Iacobuzio-Donahue, Zachary Kerner, Stephen B. Baylin, Christopher L. Wolfgang, Nita Ahuja

**Affiliations:** ^1^ Department of Gastrointestinal Surgery II, Key Laboratory of Hubei Province for Digestive System Disease, Renmin Hospital, Wuhan University, Wuhan 430060, China; ^2^ Department of Colon and Rectal Surgery, Columbia University Medical Center, New York, NY 10032, USA; ^3^ Department of Pathology and David Rubenstein Pancreatic Cancer Research Center, Memorial Sloan Kettering Cancer Center, New York, NY 10065, USA; ^4^ Department of Surgery, The Johns Hopkins University School of Medicine, Baltimore, MD 21287, USA; ^5^ Department of Oncology, The Johns Hopkins University School of Medicine, Baltimore, MD 21287, USA; ^6^ Department of Urology, The Johns Hopkins University School of Medicine, Baltimore, MD 21287, USA

**Keywords:** CpG island methylator phenotype, MLH1, methylation, colorectal cancer, prognosis

## Abstract

We previously developed a novel tumor subtype classification model for duodenal adenocarcinomas based on a combination of the CpG island methylator phenotype (CIMP) and *MLH1* methylation status. Here, we tested the prognostic value of this model in stage II colorectal cancer (CRC) patients. Tumors were assigned to CIMP+/*MLH1*-unmethylated (*MLH1*-U), CIMP+/*MLH1*-methylated (*MLH1*-M), CIMP−/*MLH1*-U, or CIMP−/*MLH1*-M groups. Age, tumor location, lymphovascular invasion, and mucin production differed among the four patient subgroups, and CIMP+/*MLH1*-U tumors were more likely to have lymphovascular invasion and mucin production. Kaplan-Meier analyses revealed differences in both disease-free survival (DFS) and overall survival (OS) among the four groups. In a multivariate analysis, CIMP/*MLH1* methylation status was predictive of both DFS and OS, and DFS and OS were shortest in CIMP+/*MLH1*-U stage II CRC patients. These results suggest that tumor subtype classification based on the combination of CIMP and *MLH1* methylation status is informative in stage II CRC patients, and that CIMP+/*MLH1*-U tumors exhibit aggressive features and are associated with poor clinical outcomes.

## INTRODUCTION

Colorectal cancer (CRC) is the third most common cause of cancer-related death in the United States [1], and an estimated 134,490 new cases will be diagnosed in 2016 [2]. The 5-year relative survival rate for CRC patients is 65% [2]. The 5-year relative survival rate for stage II CRCs is about 82.5% [3]; thus, approximately 20% of patients with tumors of this stage ultimately die of recurrent disease. Currently, clinical and pathologic stage are the primary bases for selecting therapies for CRC patients. Patients with stage I disease have an excellent prognosis after surgery alone (80% to 95% 5-year survival) [4], while adjuvant chemotherapy improves both disease-free and overall disease-specific survival in patients with stage III disease [5]. However, the use of chemotherapy after curative operation remains controversial in stage II colorectal cancer patients. Stage II CRC patients are generally considered to be at low risk of postoperative recurrence; therefore, routine adjuvant chemotherapy is generally not recommended for these patients [6]. However, a recent study found that stage IIIa CRC patients who received chemotherapy survived longer than stage IIb patients who did not [3]. Identification of reliable prognostic molecular markers for stage II CRC patients is therefore crucial. The ability of current histopathology- and imaging-based staging to predict CRC prognosis is limited, suggesting that molecular classifications should be refined [7].

Defining cancer subtypes based on pathway-driven alterations has the potential to improve prognoses. The chromosomal instability (CIN), microsatellite instability (MSI), and CpG island methylator phenotype (CIMP) pathways play important roles in CRC tumorigenesis. Although CIN contributes to the majority of CRCs, approximately 15% of tumors develop via MSI [8]. MSI is the abnormal shortening or lengthening of DNA by 1 to 6 repeating base pair units resulting from deficient DNA mismatch repair (dMMR). dMMR can result from germline mutations in MMR genes (*MLH1*, *MSH2*, *MSH6*, and *PMS2*), i.e., Lynch syndrome [9]. However, most instances of dMMR in CRC are due to sporadic epigenetic inactivation of *MLH1* [10]. Methylation of *MLH1* is also a major molecular determinant of CIMP-positive (CIMP+) CRC and is often included in the traditional CIMP markers panel; however, *MLH1* methylation-associated features have not been investigated fully in CIMP+ CRC [11]. The CIMP classification was first proposed in 1999 to describe a subset of CRCs with aberrant methylation of cytosine residues at CpG islands in the promoter regions of multiple cancer-specific genes [12]. From then on, various methylation markers have been developed and used to determine CIMP status in CRC tumors, but these results have been inconsistent and arbitrary. The classic CIMP panel consists of five CIMP markers: *CDKN2A*, *MINT1*, *MINT2*, *MINT31*, and *MLH1*. Weisenberger *et al*. developed a new five-marker panel consisting of *CACNA1G*, *IGF2*, *NEUROG1*, *RUNX3*, and *SOCS1*, and found that it was more accurate in determining CIMP status than the classic panel [13]. Interestingly, *MLH1* methylation has also been detected in some CIMP negative (CIMP−) tumors, and *MLH1-*unmethylated (*MLH1-*U) tumors can be found in CIMP+ CRCs [12, 13]. We recently developed a model that combines CIMP makers and *MLH1* methylation status to stratify tumor subtypes and found that it may assist in determining prognosis in duodenal adenocarcinoma patients. Patients with CIMP+/*MLH1-*U tumors had the worst prognoses [14]. In a more recent study, Kim *et al*. found that CIMP+ status in both *MLH1*-methylated (*MLH1-*M) and *MLH1*-U CRC patients was associated with adverse clinicopathologic and molecular features [11].

In this study, we examined associations between tumor subtypes as defined by CIMP/*MLH1* methylation status and clinicopathologic and molecular characteristics, and explored the impact of these subtypes on clinical outcomes. These data may provide further insight into the clinical behavior of CIMP+/*MLH1*-U tumors versus other tumor subtypes and may be useful in patient management and clinical decision-making.

## RESULTS

### Associations between CIMP and *MLH1* methylation status and clinicopathologic characteristics

DNA extraction and CIMP testing using MethyLight were successful in all 115 patients. Twenty-five (21.7%) of the 115 patients tested were CIMP+. Among the CIMP+ tumors, 9 (36.0%) were *MLH1*-U and 16 (64.0%) were *MLH1*-M. Based on CIMP and *MLH1* methylation status, tumors were assigned to CIMP+/*MLH1*-U (*n* = 9), CIMP+/*MLH1*-M (*n* = 16), CIMP−/*MLH1*-U (*n* = 77), and CIMP−/*MLH1*-M (*n* = 13) groups. Among the clinical characteristics examined, the four patient subgroups differed in terms of age (*P* = 0.003), tumor location (*P* = 0.001), lymphovascular invasion (*P* = 0.034), and mucin production (*P* = 0.013; Table 1). Most patients with CIMP+ tumors (CIMP+/*MLH1*-U and CIMP+/*MLH1*-M groups) were 60 years old or older, especially in the CIMP+/*MLH1*-M group (93.8%). CIMP+/*MLH1*-M tumors were more likely to be proximally located (93.8%), whereas CIMP−/*MLH1*-M tumors were frequently observed in the distal colon and rectum (69.2%). However, both CIMP+/*MLH1*-U and CIMP−/*MLH1*-U tumors had diffuse distribution, with 55.6% and 44.2% located in the proximal colon, respectively (*P* = 0.001). CIMP+/*MLH1*-U tumors were more likely to involve lymphovascular invasion (55.6 versus 12.5–15.4 %; *P* = 0.034) and mucin production (55.6 versus 0–18.2%; *P* = 0.013; Table 1) than those in the other three groups; these differences remained statistically significant after examination using Fisher's exact test (*P* = 0.008 for lymphovascular invasion, *P* = 0.010 for mucin production). No correlations were found between tumor subgroup and molecular features (*KRAS* mutations, *P* = 0.512, MSI status, *P* = 0.919).

**Table 1:** Differences in clinicopathologic features of colorectal cancer depending on CIMP and *MLH1* methylation status

Variable		Total*n*(*n* = 115)	CIMP+*/MLH1-*U(*n* = 9)	CIMP+/*MLH1-*M(*n* = 16)	CIMP−*/MLH1-*U(*n* = 77)	CIMP−*/MLH1-*M(*n* = 13)	*P*-value
Age	< 60	38 (33.0)	2 (22.2)	1 (6.3)	26 (33.8)	9 (69.2)	0.003
	≥ 60	77 (67.0)	7 (77.8)	15 (93.8)	51 (66.2)	4 (30.8)	
Sex	Male	52 (45.2)	4 (44.4)	7 (43.8)	37 (48.1)	4 (30.8)	0.735
	Female	63 (54.8)	5 (55.6)	9 (56.3)	40 (51.9)	9 (69.2)	
Location	Proximal	58 (50.4)	5 (55.6)	15 (93.8)	34 (44.2)	4 (30.8)	0.001
	Distal	57 (49.6)	4 (44.4)	1 (6.3)	43 (55.8)	9 (69.2)	
Lymph nodes examined	≥ 12	80 (69.6)	7 (77.8)	13 (81.3)	49 (63.6)	11 (84.6)	0.236
	< 12	35 (30.4)	2 (22.2)	3 (18.8)	28 (36.4)	2 (15.4)	
Differentiation	Well to moderate	95 (82.6)	6 (66.7)	12 (75.0)	66 (85.7)	11 (84.6)	0.363
	Poor	20 (17.4)	3 (33.3)	4 (25.0)	11 (14.3)	2 (15.4)	
pT4	No	103 (89.6)	8 (88.9)	16 (100)	68 (88.3)	11 (84.6)	0.496
	Yes	12 (10.4)	1 (11.1)	0 (0)	9 (11.7)	2 (15.4)	
Lymphovascular invasion	No	95 (82.6)	4 (44.4)	14 (87.5)	66 (85.7)	11 (84.6)	0.034
	Yes	20 (17.4)	5 (55.6)	2 (12.5)	11 (14.3)	2 (15.4)	
Mucin production	No	94 (81.7)	4 (44.4)	14 (87.5)	63 (81.8)	13 (100.0)	0.013
	Yes	21 (18.3)	5 (55.6)	2 (12.5)	14 (18.2)	0 (0)	
*KRAS*	Wild type	75 (65.2)	4 (44.4)	10 (62.5)	53 (68.8)	8 (61.5)	0.512
	Mutant	40 (34.8)	5 (55.6)	6 (37.5)	24 (31.2)	5 (38.5)	
MSI status	MSS + MSI-low	59 (51.3)	5 (55.6)	7 (43.8)	40 (51.9)	7 (53.8)	0.919
	MSI-high	56 (48.7)	4 (44.4)	9 (56.3)	37 (48.1)	6 (46.2)	

Abbreviations: CIMP+, CpG island methylator phenotype positive; CIMP−, CpG island methylator phenotype negative; *MLH1*-U, *MLH1*-unmethylated; *MLH1*-M, *MLH1-*methylated; MSI, microsatellite instability

### Survival is poorest in CRC patients with CIMP+/*MLH1*-U tumors

**Figure 1:**
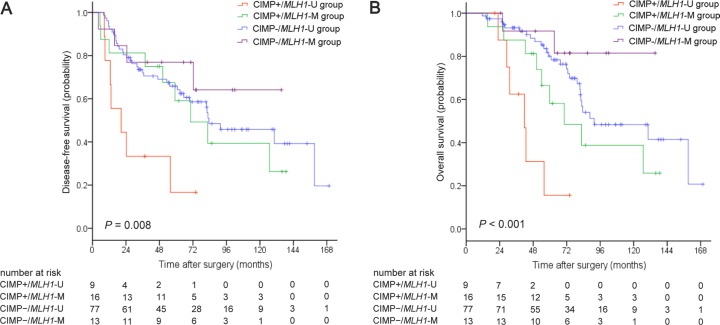
Kaplan-Meier survival estimates in stage II colorectal cancer patient groups classified by CIMP/*MLH1* methylation status (**A**) Disease-free survival, (**B**) Overall survival. *P* values were calculated using the log-rank test.

**Figure 2:**
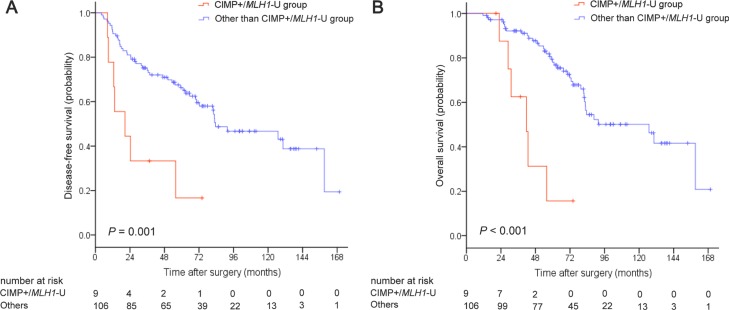
Kaplan-Meier survival estimates in stage II colorectal cancer patients with CIMP+/*MLH1*-unmethylated tumors compared to the remaining three patient groups (**A**) Disease-free survival, (**B**) Overall survival. *P* values were calculated using the log-rank test.

Kaplan-Meier survival curves were generated based on CIMP and *MLH1* methylation status. For the overall patient cohort, median disease-free survival (DFS) and overall survival (OS) were 82.6 months (95% CI, 64.0–101.2) and 88.6 months (95% CI, 42.5–134.7), respectively. Five-year DFS and OS rates were 62.6% and 73.6%, respectively. Both DFS (*P* = 0.008; Figure 1A) and OS (*P <* 0.001; Figure 1B) differed among the four CIMP/*MLH1* methylation subgroups. Median DFS and OS were 20.6 months (95% CI, 0–41.9) and 41.7 months (95% CI, 28.0–55.4) for the CIMP+/*MLH1*-U group, 70.3 months (95% CI, 37.1–103.5) and 70.3 months (95% CI, 37.6–103.0) for the CIMP+/*MLH1*-M group, and 83.5 months (95% CI, 45.1–121.9) and 91.6 months (95% CI, 45.1–138.1) for the CIMP−/*MLH1*-U group; median DFS and OS could not be reached for the CIMP−/*MLH1*-M group. DFS and OS were shorter for CIMP+/*MLH1*-U group patients than for patients in each of the other three groups (versus the CIMP+/*MLH1*-M group: *P* = 0.046 for DFS, *P* = 0.046 for OS; versus the CIMP−/*MLH1*-U group: *P* = 0.001 for DFS, *P <* 0.001 for OS; versus the CIMP−/*MLH1*-M group: *P* = 0.016 for DFS, *P* = 0.003 for OS). DFS and OS were also dramatically shorter in patients with CIMP+/*MLH1*-U tumors compared to the other three patient groups combined (*P* = 0.001 for DFS, *P <* 0.001 for OS, respectively; Figure 2A, 2B).

### Univariate and multivariate analyses of outcome predictors

Next, we conducted univariate and multivariate analyses using a Cox proportional hazards model to examine associations between sex, number of lymph nodes examined, pT4, lymphovascular invasion, tumor location, differentiation, *KRAS* mutations, MSI, and CIMP/*MLH1* status (CIMP+/*MLH1*-U or other subtypes) and DFS and OS (Table 2). In univariate analysis, CIMP+/*MLH1*-U status was a predictor of shorter DFS (*P* = 0.002; hazard ratio (HR) = 3.59, 95% CI, 1.59–8.08) and OS (*P* = 0.000; HR = 5.49, 95% CI, 2.21–13.62); lymphovascular invasion also predicted shorter OS (*P* = 0.047; HR = 2.13, 95% CI, 1.01–4.52). In multivariate analysis, only CIMP+/*MLH1*-U status independently predicted DFS (*P* = 0.011; HR = 3.16, 95% CI, 1.30–7.64) after adjusting for age, number of lymph nodes examined, pT4, and lymphovascular invasion and OS (*P* = 0.002; HR = 4.70, 95% CI, 1.74–12.72) after adjusting for age and lymphovascular invasion.

**Table 2:** Univariate and multivariate Cox proportional hazard analysis of disease-free survival and overall survival

Variable	Total*n*	DFS				OS			
		Univariate		Multivariate		Univariate		Multivariate	
		HR (95% CI)	*P* value	HR (95% CI)	*P* value	HR (95% CI)	*P* value	HR (95% CI)	*P* value
Age			0.249		0.262		0.152		0.100
< 60	38	1.00 (Reference)		1.00 (Reference)		1.00 (Reference)		1.00 (Reference)	
≥ 60	77	1.42 (0.78–2.57)		1.40 (0.77–2.57)		1.65 (0.83–3.26)		1.78 (0.90–3.54)	
Sex			0.396				0.958		
Male	52	1.00 (Reference)				1.00 (Reference)			
Female	63	1.26 (0.74–2.12)				0.98 (0.55–1.77)			
Location			0.948				0.449		
Proximal	58	1.00 (Reference)				1.00 (Reference)			
Distal	57	1.02 (0.60–1.72)				0.80 (0.44–1.43)			
Lymph nodes examined			0.177		0.108		0.590		
≥ 12	80	1.00 (Reference)		1.00 (Reference)		1.00 (Reference)			
< 12	35	1.46 (0.84–2.52)		1.61 (0.90–2.88)		1.19 (0.64–2.20)			
Differentiation			0.415				0.670		
Well to moderate	95	1.00 (Reference)				1.00 (Reference)			
Poor	20	0.73 (0.35–1.55)				0.84 (0.37–1.88)			
pT4			0.262		0.456		0.378		
No	103	1.00 (Reference)		1.00 (Reference)		1.00 (Reference)			
Yes	12	0.56 (0.20–1.55)		0.67 (0.23–1.92)		0.63 (0.23–1.76)			
Lymphovascular invasion			0.101		0.193		0.047		0.200
No	95	1.00 (Reference)		1.00 (Reference)		1.00 (Reference)		1.00 (Reference)	
Yes	20	1.76(0.90–3.44)		1.64 (0.78–3.43)		2.13 (1.01–4.52)		1.73 (0.75–3.99)	
Mucin production			0.422				0.466		
No	94	1.00 (Reference)				1.00 (Reference)			
Yes	21	1.29 (0.69–2.41)				1.29 (0.65–2.56)			
*KRAS*			0.524				0.708		
Wild type	75	1.00 (Reference)				1.00 (Reference)			
Mutant	40	1.20 (0.69–2.07)				1.13 (0.61–2.08)			
MSI status			0.470				0.419		
MSS+MSI-low	59	1.00 (Reference)				1.00 (Reference)			
MSI-high	56	0.82 (0.48–1.40)				0.78 (0.43–1.42)			
CIMP/*MLH1*			0.002		0.011		0.000		0.002
Others	106	1.00 (Reference)		1.00 (Reference)		1.00 (Reference)		1.00 (Reference)	
CIMP+/*MLH1***-**U	9	3.59 (1.59–8.08)		3.16 (1.30–7.64)		5.49 (2.21–13.62)		4.70 (1.74–12.72)	

Abbreviations: DFS, disease-free survival; OS, overall survival; HR, hazard ratio; CI, confidence interval; CIMP, CpG island methylator phenotype; *MLH1*-M, *MLH1*-methylated; *MLH1*-U, *MLH1*-unmethylated; MSS, microsatellite stable; MSI, microsatellite instability. A backward elimination with threshold of *P* = 0.300 was used to select variables in the final models.

## DISCUSSION

In this study, we examined differences in survival in a cohort of stage II CRC patients with different genetic and epigenetic tumor profiles. We found that classifying these tumors based on CIMP and *MLH1* methylation status created more uniform clinical CRC subsets that share common genetic features. Additionally, CIMP+/*MLH1*-U tumor status was associated with highly aggressive disease and poor prognosis in stage II CRC patients. Routine identification and further characterization of this tumor subtype might help to optimize CRC therapies and identify patients who may benefit from additional therapies.

Recently developed molecular classification systems will likely improve the stratification and treatment of CRC patients [7, 15–18]. In a previous study of the largest cohort of duodenal adenocarcinoma patients to date, we classified tumors into four subtypes based on the combination of CIMP and *MLH1* methylation status. The addition of this *MLH1* methylation status biomarker improved predictions of OS and time to recurrence in CIMP+ duodenal adenocarcinoma patients [14]. Furthermore, in a recent study of CIMP+ CRCs, OS decreased in *MLH1*-U patients compared to *MLH1*-M patients [19]. Here, we found that clinicopathologic features and survival rates also differed among stage II CRC patients assigned to different subgroups based on CIMP/*MLH1* methylation status.

CIMP−/*MLH1*-U tumors were the most common subtype, representing 67% of the study cohort. However, CIMP+ tumors, and especially CIMP+/*MLH1*-M tumors (> 90 %), were more common than CIMP− tumors in older stage II CRC patients (≥ 60). A previous study revealed that CIMP+ tumors tend to have proximal locations [13]. Here, more than 90% of CIMP+/*MLH1*-M tumors were located in the proximal colon, whereas approximately 70% of CIMP−/*MLH1*-M tumors were located in the distal colon or rectum. However, CIMP+/*MLH1*-U tumors and CIMP−/*MLH1*-U tumors were evenly distributed in terms of proximal and distal location.

Both lymphovascular invasion and mucinous histology can be used to identify aggressive tumors and are indicative of unfavorable outcomes in CRC patients [20–22]. For example, lymphovascular invasion is associated with a high risk of lymph node metastasis even in T1 CRC patients [23]. Moreover, previous animal and clinical experiments have shown that increased production of mucin in human colon cancer cells correlates with increases in their metastatic potential and ability to colonize the liver [24–26]. Mucin production is also associated with CIMP+, MSI-H, and *BRAF* mutations in CRC [27]. Here, we found that CIMP+/*MLH1*-U tumors were associated with increased lymphovascular invasion and mucin production, which occurred in more than half of CIMP+/*MLH1*-U tumors and in less than one-fifth of tumors in the other groups. These findings suggest that CIMP+/*MLH1*-U status might identify a unique class of highly aggressive CRCs.

CIMP and *MLH1* methylation status are also associated with clinicopathologic and genetic differences in CRC as shown by Kim and colleagues [11]. In that study, CIMP+/*MLH1*-M tumors were associated with old age and proximal colonic tumor locations, while CIMP+/*MLH1*-U tumors were associated with vascular invasion, which is consistent with our results. However, conflicting data regarding associations between CIMP+/*MLH1*-M status and mucinous histology and between CIMP+/*MLH1*-U status and *KRAS* mutations have been reported; these associations were not detected in this study. These discrepancies may be explained by differences in patient populations, inclusion of different tumor stages, details of follow-up, and other factors associated with prognosis (such as chemotherapy and/or radiotherapy).

Analysis of individual biomarkers provides valuable predictive and prognostic information. Among the commonly analyzed biomarkers, MSI has been studied most extensively and is associated with survival in CRC patients [28–30]. A meta-analysis revealed that MSI-H CRCs were associated with a 40% improvement in OS compared to MSS tumors (95% CI, 0.31–0.47) [30]. The MSI-H tumor rate (48.7%) was relatively higher here than in previous studies; this difference might be explained at least in part by differences in inclusion criteria. Although the prognostic value of CIMP status in CRC has been inconsistent [31–35], a meta-analysis concluded that CIMP+ status was associated with poor prognosis in both MSI-high and MSS+MSI-low tumors [35]. Multiple studies support the feasibility of combining molecular markers to classify CRCs [32, 36]. Two studies in large population-based cohorts of individuals from the Colon Cancer Family Registry investigated associations between CIMP status and molecular features, risk factors, family history, and survival [37, 38]. Weisenberger *et al*. found that CIMP status was associated with MSI-H, *BRAF* mutation, proximal tumor sites, female patients, older age, and family history of CRC [37]. Furthermore, Phipps *et al*. found that survival differed among tumor subtypes defined by the combination of MSI, CIMP, *BRAF* mutation, and *KRAS* mutation status, which was derived from the classifications proposed by Samadder *et al*. [38, 39]. Patients with MSI-H disease subtypes (i.e., MSI-H, CIMP+, *BRAF* mutant, and *KRAS* wild-type tumors, and MSI-H, CIMP−, *BRAF* wild-type, and *KRAS* wild-type tumors) survived longest, and those with MSS or MSI-L, CIMP+, *BRAF* mutant, and *KRAS* wild-type tumors had the highest mortality [38]. However, this cohort included tumors of all stages, and the prognostic value of these classifications in stage II tumors requires further investigation.

Because the stage II CRC patients included in this study did not receive neoadjuvant chemotherapy, our survival analysis was not subject to potentially confounding prognostic factors related to this treatment. Among the four CIMP/*MLH1* methylation status groups, DFS and OS were shorter in the CIMP+/*MLH1*-U group than in the other three groups separately or combined. Most importantly, CIMP+/*MLH1*-U status was the only factor that independently predicted both DFS and OS after adjusting for other factors. Our findings, together with previous studies, demonstrate that novel subtype classifications may help to identify aggressive tumors and predict clinical outcomes.

The mechanisms by which *MLH1* methylation affects progression in stage II CRC remain unclear. MSI allows mutations to accumulate rapidly and facilitates tumor progression [40]; this high mutational load in MSI tumors typically creates 10 to 50 times more tumor-specific neoantigens than are found in MSS tumors [41]. Additionally, abnormalities in Human Leukocyte Antigen (HLA) class I expression are common in CRCs. Since HLA expression is required for the activation of tumor antigen-specific cytotoxic T-lymphocytes (CTL), HLA class I abnormalities allow tumors to circumvent immune surveillance. The ability to evade CTL activity may be especially important for MSI-H tumors, since they produce large amounts of neoantigens. Previous studies have demonstrated that HLA class I alterations are present in MSI-H tumors. For example, Dierssen *et al*. showed that distinct mechanisms are responsible for HLA class I loss in different tumor subtypes; loss of HLA class 1 expression was associated with the loss of β2m in HNPCC tumors and with APM component defects in MSI-H tumors [42]. Most sporadic, MSI-H CRC tumors are associated with alterations in *MLH1* methylation. Most strikingly, Scarpa *et al*. showed that patients with sporadic, MSI-H CRC had higher T-bet/CD4 ratios, CD80 expression rates, and CD8 lymphocyte infiltration compared to those with MSS tumors. Moreover, the Treg marker FoxP-3 was expressed in the MSS group, but not in the MSI-H group. Additionally, survival was better in stage I and II MSI-H patients with T-bet expression. Furthermore, *in vitro* experiments revealed that CD80 expression was higher in the HTC-15 cell line, which has a MSI-H status, than in the HT-29 MSS colon cancer cell line. When *MLH1* expression was inhibited in HT-29 cells, numbers of CD80+ cells increased [43]. Based on these results, we speculate that the importance of *MLH1* methylation for tumor classification may be due to variation in the causes of methylation in many cancer-specific genes, including *MLH1*, and to the distinct mechanisms underlying loss of HLA class I expression and enhanced immune surveillance in *MLH1*-M tumors.

In conclusion, the current results validate our novel classification strategy that uses CIMP and *MLH1* methylation status in combination to determine tumor subtypes in stage II CRC patients. CIMP+/*MLH1*-U tumors were the most aggressive and were associated with the poorest clinical outcomes, suggesting that new adjuvant therapies should be developed for this patient subgroup. Future investigations of the mechanisms underlying these associations will help improve the understanding of CRC tumorigenesis.

## MATERIALS AND METHODS

### Study population

This retrospective cohort study included patients with pathologically confirmed stage II (T1-4N0M0) CRC tumors who underwent radical resections at the Johns Hopkins Bayview Hospital (JHBH) and the Johns Hopkins Hospital (JHH) between 1995 and 2009. Patients who received neoadjuvant chemotherapy or for whom follow-up information, archival primary tumors, or corresponding matched normal samples were missing were excluded from the study. Formalin-fixed, paraffin-embedded (FFPE) colorectal cancer tissue and adjacent non-neoplastic colorectal tissue samples from 115 stage II colorectal cancer patients were obtained from JHBH and JHH with approval from the Institutional Review Board (IRB) and in accordance with Health Insurance Portability and Accountability Act (HIPAA) regulations. The histopathology of each specimen was examined to confirm the diagnosis.

### *KRAS* mutation and MSI analysis

Genomic DNA from FFPE tissue was extracted using phenol chloroform, and polymerase chain reaction (PCR) targeting *KRAS* codons 12 and 13 was performed as previously described [44]. PCR products were sequenced in both directions using M13F (5′-GTAAAACGACGGCCAGT-3′) and R (5′-CAGGAAACAGCTATGACC-3′) primers (Agencourt Bioscience Corporation). Sequence data were analyzed using Sequencher 4.8 software (Gene Codes). All mutations were verified using bidirectional sequencing of a second PCR product derived independently from the original template.

MSI status was determined by examining D2S123, D5S346, D17S250, BAT25, and BAT26 [45]. Microsatellite sizes were compared with those of normal adjacent tissue, and tumors with instability in 2 or more of the markers were classified as high MSI (MSI-H). Tumors with instability in only 1 marker or without instability in these markers were classified as low MSI (MSI-L) or microsatellite stable (MSS), respectively.

### Bisulfite modification and methylation analysis

Purified DNA (2 mg) was bisulfite-treated and purified using the EZ DNA methylation kit (Zymo Research) according to the manufacturer's instructions. The 5-gene signature used to assess CIMP methylation status in primary tumor tissues consisted of *CACNA1G*, *IGF2*, *NEUROG1*, *RUNX3*, and *SOCS1* [13]. Methylation was quantified using MethyLight, a methylation-specific, probe based, real-time PCR technique [13, 46]. Alu was used as a normalization control reaction. All CIMP probes used a 5′ FAM fluorophore, a 3′ IBFQ quencher, and an internal ZEN quencher (Integrated DNA Technologies) [14]. DNA methylation is reported as percent of methylated reference (PMR) = 100 × [(methylated reaction/Alu)_sample_/(methylated reaction/Alu)_M.SssI-reference_)]. Markers were considered methylated when PMR = 10. Samples were considered CIMP+ if at least 3 of the 5 studied genes were methylated [13].

### Statistical analysis

The primary clinical end point was DFS, which was measured from the date of surgery to the date of any event, regardless of cause [47]. Date of recurrence was established by radiographic studies, laboratory studies, physical examination, and/or histopathology. OS, defined as the time from surgery to death irrespective of cause, was a secondary outcome [47]. Statistical analysis was performed using SPSS software (version 18.0; SPSS, Chicago, IL). Clinicopathologic factors were compared using *χ2* tests with the Monte Carlo method or Fisher's exact test. Results were considered significant at *P <* 0.05. Survival was estimated using the Kaplan-Meier method and log-rank test. Cox proportional hazard regression models were used to determine univariate and multivariate HRs.

## References

[1] Siegel RL, Miller KD, Jemal A (2016). Cancer statistics, 2016. Cancer J Clin.

[2] Miller KD, Siegel RL, Lin CC, Mariotto AB, Kramer JL, Rowland JH, Stein KD, Alteri R, Jemal A (2016). Cancer treatment and survivorship statistics, 2016. Cancer J Clin.

[3] O'Connell JB, Maggard MA, Ko CY (2004). Colon cancer survival rates with the new American Joint Committee on Cancer sixth edition staging. J Natl Cancer Inst.

[4] Galandiuk S, Wieand HS, Moertel CG, Cha SS, Fitzgibbons RJ, Pemberton JH, Wolff BG (1992). Patterns of recurrence after curative resection of carcinoma of the colon and rectum. Surg Gynecol Obstet.

[5] Moertel CG (1992). Accomplishments in surgical adjuvant therapy for large bowel cancer. Cancer.

[6] NIH consensus conference (1990). Adjuvant therapy for patients with colon and rectal cancer. Jama.

[7] Calon A, Lonardo E, Berenguer-Llergo A, Espinet E, Hernando-Momblona X, Iglesias M, Sevillano M, Palomo-Ponce S, Tauriello DV, Byrom D, Cortina C, Morral C, Barcelo C (2015). Stromal gene expression defines poor-prognosis subtypes in colorectal cancer. Nat Genet.

[8] Sinicrope FA, Foster NR, Thibodeau SN, Marsoni S, Monges G, Labianca R, Kim GP, Yothers G, Allegra C, Moore MJ, Gallinger S, Sargent DJ (2011). DNA mismatch repair status and colon cancer recurrence and survival in clinical trials of 5-fluorouracil-based adjuvant therapy. J Natl Cancer Inst.

[9] Vasen HF, Watson P, Mecklin JP, Lynch HT (1999). New clinical criteria for hereditary nonpolyposis colorectal cancer (HNPCC, Lynch syndrome) proposed by the International Collaborative group on HNPCC. Gastroenterology.

[10] Herman JG, Umar A, Polyak K, Graff JR, Ahuja N, Issa JP, Markowitz S, Willson JK, Hamilton SR, Kinzler KW, Kane MF, Kolodner RD, Vogelstein B (1998). Incidence and functional consequences of hMLH1 promoter hypermethylation in colorectal carcinoma. Proc Natl Acad Sci USA.

[11] Kim JH, Bae JM, Cho NY, Kang GH (2016). Distinct features between MLH1-methylated and unmethylated colorectal carcinomas with the CpG island methylator phenotype: implications in the serrated neoplasia pathway. Oncotarget.

[12] Toyota M, Ahuja N, Ohe-Toyota M, Herman JG, Baylin SB, Issa JP (1999). CpG island methylator phenotype in colorectal cancer. Proc Natl Acad Sci U S A.

[13] Weisenberger DJ, Siegmund KD, Campan M, Young J, Long TI, Faasse MA, Kang GH, Widschwendter M, Weener D, Buchanan D, Koh H, Simms L, Barker M (2006). CpG island methylator phenotype underlies sporadic microsatellite instability and is tightly associated with BRAF mutation in colorectal cancer. Nat Genet.

[14] Fu T, Pappou EP, Guzzetta AA, Jeschke J, Kwak R, Dave P, Hooker CM, Morgan R, Baylin SB, Iacobuzio-Donahue CA, Wolfgang CL, Ahuja N (2012). CpG island methylator phenotype-positive tumors in the absence of MLH1 methylation constitute a distinct subset of duodenal adenocarcinomas and are associated with poor prognosis. Clin Cancer Res.

[15] Cleven AH, Derks S, Draht MX, Smits KM, Melotte V, Van Neste L, Tournier B, Jooste V, Chapusot C, Weijenberg MP, Herman JG, de Bruine AP, van Engeland M (2014). CHFR promoter methylation indicates poor prognosis in stage II microsatellite stable colorectal cancer. Clin Cancer Res.

[16] Draht MX, Smits KM, Tournier B, Jooste V, Chapusot C, Carvalho B, Cleven AH, Derks S, Wouters KA, Belt EJ, Stockmann HB, Bril H, Weijenberg MP (2014). Promoter CpG island methylation of RET predicts poor prognosis in stage II colorectal cancer patients. Mol Oncol.

[17] De Sousa EMF, Wang X, Jansen M, Fessler E, Trinh A, de Rooij LP, de Jong JH, de Boer OJ, van Leersum R, Bijlsma MF, Rodermond H, van der Heijden M, van Noesel CJ (2013). Poor-prognosis colon cancer is defined by a molecularly distinct subtype and develops from serrated precursor lesions. Nat Med.

[18] Saito T, Nishikawa H, Wada H, Nagano Y, Sugiyama D, Atarashi K, Maeda Y, Hamaguchi M, Ohkura N, Sato E, Nagase H, Nishimura J, Yamamoto H (2016). Two FOXP3(+)CD4(+) T cell subpopulations distinctly control the prognosis of colorectal cancers. Nat Med.

[19] Levine AJ, Phipps AI, Baron JA, Buchanan DD, Ahnen DJ, Cohen SA, Lindor NM, Newcomb PA, Rosty C, Haile RW, Laird PW, Weisenberger DJ (2016). Clinicopathologic Risk Factor Distributions for MLH1 Promoter Region Methylation in CIMP-Positive Tumors. Cancer Epidemiol Biomarkers Prev.

[20] Chang SC, Lin PC, Lin JK, Lin CH, Yang SH, Liang WY, Chen WS, Jiang JK (2016). Mutation Spectra of Common Cancer-Associated Genes in Different Phenotypes of Colorectal Carcinoma Without Distant Metastasis. Ann Surg Oncol.

[21] Kim SH, Shin SJ, Lee KY, Kim H, Kim TI, Kang DR, Hur H, Min BS, Kim NK, Chung HC, Roh JK, Ahn JB (2013). Prognostic value of mucinous histology depends on microsatellite instability status in patients with stage III colon cancer treated with adjuvant FOLFOX chemotherapy: a retrospective cohort study. Ann Surg Oncol.

[22] Huh JW, Lee JH, Kim HR, Kim YJ (2013). Prognostic significance of lymphovascular or perineural invasion in patients with locally advanced colorectal cancer. Am J Surg.

[23] Nascimbeni R, Burgart LJ, Nivatvongs S, Larson DR (2002). Risk of lymph node metastasis in T1 carcinoma of the colon and rectum. Dis Colon Rectum.

[24] Bresalier RS, Niv Y, Byrd JC, Duh QY, Toribara NW, Rockwell RW, Dahiya R, Kim YS (1991). Mucin production by human colonic carcinoma cells correlates with their metastatic potential in animal models of colon cancer metastasis. J Clin Invest.

[25] Niv Y (1994). Mucin and colorectal cancer metastasis. Am J Gastroenterol.

[26] Khanh do T, Mekata E, Mukaisho K, Sugihara H, Shimizu T, Shiomi H, Murata S, Naka S, Yamamoto H, Endo Y, Tani T (2013). Transmembrane mucin MUC1 overexpression and its association with CD10(+) myeloid cells, transforming growth factor-beta1 expression, and tumor budding grade in colorectal cancer. Cancer Sci.

[27] Tanaka H, Deng G, Matsuzaki K, Kakar S, Kim GE, Miura S, Sleisenger MH, Kim YS (2006). BRAF mutation, CpG island methylator phenotype and microsatellite instability occur more frequently and concordantly in mucinous than non-mucinous colorectal cancer. Int J Cancer.

[28] Mouradov D, Domingo E, Gibbs P, Jorissen RN, Li S, Soo PY, Lipton L, Desai J, Danielsen HE, Oukrif D, Novelli M, Yau C, Holmes CC (2013). Survival in stage II/III colorectal cancer is independently predicted by chromosomal and microsatellite instability, but not by specific driver mutations. Am J Gastroenterol.

[29] Merok MA, Ahlquist T, Royrvik EC, Tufteland KF, Hektoen M, Sjo OH, Mala T, Svindland A, Lothe RA, Nesbakken A (2013). Microsatellite instability has a positive prognostic impact on stage II colorectal cancer after complete resection: results from a large, consecutive Norwegian series. Ann Oncol.

[30] Guastadisegni C, Colafranceschi M, Ottini L, Dogliotti E (2010). Microsatellite instability as a marker of prognosis and response to therapy: a meta-analysis of colorectal cancer survival data. Eur J Cancer.

[31] Cha Y, Kim KJ, Han SW, Rhee YY, Bae JM, Wen X, Cho NY, Lee DW, Lee KH, Kim TY, Oh DY, Im SA, Bang YJ (2016). Adverse prognostic impact of the CpG island methylator phenotype in metastatic colorectal cancer. Br J Cancer.

[32] Ogino S, Nosho K, Kirkner GJ, Kawasaki T, Meyerhardt M, Loda JA, Giovannucci EL, Fuchs CS (2009). CpG island methylator phenotype, microsatellite instability, BRAF mutation and clinical outcome in colon cancer. Gut.

[33] Barault L, Charon-Barra C, Jooste V, de la Vega MF, Martin L, Roignot P, Rat P, Bouvier AM, Laurent-Puig P, Faivre J, Chapusot C, Piard F (2008). Hypermethylator phenotype in sporadic colon cancer: study on a population-based series of 582 cases. Cancer Res.

[34] Jia M, Gao X, Zhang Y, Hoffmeister M, Brenner H (2016). Different definitions of CpG island methylator phenotype and outcomes of colorectal cancer: a systematic review. Clin Epigenetics.

[35] Juo YY, Johnston FM, Zhang DY, Juo HH, Wang H, Pappou EP, Yu T, Easwaran H, Baylin S, van Engeland M, Ahuja N (2014). Prognostic value of CpG island methylator phenotype among colorectal cancer patients: a systematic review and meta-analysis. Ann Oncol.

[36] Sinicrope FA, Shi Q, Smyrk TC, Thibodeau SN, Dienstmann R, Guinney J, Bot BM, Tejpar S, Delorenzi M, Goldberg RM, Mahoney M, Sargent DJ, Alberts SR (2015). Molecular markers identify subtypes of stage III colon cancer associated with patient outcomes. Gastroenterology.

[37] Weisenberger DJ, Levine AJ, Long TI, Buchanan DD, Walters R, Clendenning M, Rosty C, Joshi AD, Stern MC, Le Marchand L, Lindor NM, Daftary D, Gallinger S (2015). Association of the colorectal CpG island methylator phenotype with molecular features, risk factors, and family history. Cancer Epidemiol Biomarkers Prev.

[38] Phipps AI, Limburg PJ, Baron JA, Burnett-Hartman AN, Weisenberger DJ, Laird PW, Sinicrope FA, Rosty C, Buchanan DD, Potter JD, Newcomb PA (2015). Association between molecular subtypes of colorectal cancer and patient survival. Gastroenterology.

[39] Samadder NJ, Vierkant RA, Tillmans LS, Wang AH, Weisenberger DJ, Laird PW, Lynch CF, Anderson KE, French AJ, Haile RW, Potter JD, Slager SL, Smyrk TC (2013). Associations between colorectal cancer molecular markers and pathways with clinicopathologic features in older women. Gastroenterology.

[40] Smyrk TC, Watson P, Kaul K, Lynch HT (2001). Tumor-infiltrating lymphocytes are a marker for microsatellite instability in colorectal carcinoma. Cancer.

[41] Llosa NJ, Cruise M, Tam A, Wicks EC, Hechenbleikner EM, Taube JM, Blosser RL, Fan H, Wang H, Luber BS, Zhang M, Papadopoulos N, Kinzler KW (2015). The vigorous immune microenvironment of microsatellite instable colon cancer is balanced by multiple counter-inhibitory checkpoints. Cancer Discov.

[42] Dierssen JW, de Miranda NF, Ferrone S, van Puijenbroek M, Cornelisse CJ, Fleuren GJ, van Wezel T, Morreau H (2007). HNPCC versus sporadic microsatellite-unstable colon cancers follow different routes toward loss of HLA class I expression. BMC Cancer.

[43] Scarpa M, Ruffolo C, Canal F, Scarpa M, Basato S, Erroi F, Fiorot A, Dall'Agnese L, Pozza A, Porzionato A, Castagliuolo I, Dei Tos AP, Bassi N (2015). Mismatch repair gene defects in sporadic colorectal cancer enhance immune surveillance. Oncotarget.

[44] Yachida S, Mudali S, Martin SA, Montgomery EA, Iacobuzio-Donahue CA (2009). Beta-catenin nuclear labeling is a common feature of sessile serrated adenomas and correlates with early neoplastic progression after BRAF activation. Am J Surg Pathol.

[45] Dietmaier W, Wallinger S, Bocker T, Kullmann F, Fishel R, Ruschoff J (1997). Diagnostic microsatellite instability: definition and correlation with mismatch repair protein expression. Cancer Res.

[46] Eads CA, Danenberg KD, Kawakami K, Saltz LB, Blake C, Shibata D, Danenberg PV, Laird PW (2000). MethyLight: a high-throughput assay to measure DNA methylation. Nucleic Acids Res.

[47] Punt CJ, Buyse M, Kohne CH, Hohenberger P, Labianca R, Schmoll HJ, Pahlman L, Sobrero A, Douillard JY (2007). Endpoints in adjuvant treatment trials: a systematic review of the literature in colon cancer and proposed definitions for future trials. J Natl Cancer Inst.

